# MiR-455 targeting SOCS3 improve liver lipid disorders in diabetic mice

**DOI:** 10.1080/21623945.2020.1749495

**Published:** 2020-04-09

**Authors:** Shu Fang, Jie Feng, Hongbin Zhang, Ping Li, Yudan Zhang, Yanmei Zeng, Yingying Cai, Xiaochun Lin, Yaoming Xue, Meiping Guan

**Affiliations:** a Department of Endocrinology & Metabolism, Nanfang Hospital, Southern Medical University, Guangzhou, Guangdong, China; b Department of Medical Imaging Center, Nanfang Hospital, Southern Medical University, Guangzhou, Guangdong, China; c Department of Biomedical Sciences, University of Copenhagen, Copenhagen, Denmark; d Women and Children’s Hospital, School of Medicine, Xiamen University, Fujian, China

**Keywords:** Diabetes, NAFLD, miR-455, lipid metabolism, SOCS3

## Abstract

MiR-455 has been verified a key regulator of brown adipose tissue and adipose tissue-specific overexpression of miR-455 (ap2-miR-455) mice could combat high-fat-diet-induced obesity. This study is to verify overexpression of miR-455 could ameliorate the lipid accumulation and metabolism in the liver of db/db diabetic mice and explore the potential mechanisms. Diabetic mice (db/db) and control mice (db/m) were randomly divided into four groups. After overexpression of miR-455 in the liver of db/db mice, the triglycerides level in both serum and liver decreased, the lipid deposit in liver was improved, the expression of fatty acid synthase, stearoyl-CoA desaturase 1, sterol regulatory element binding protein 1c (SREBP-1c) and acetyl-CoA carboxylase (ACCα) was also significantly down-regulated. TargetScan indicated that suppressor of cytokine signalling 3 (SOCS3) is predicated to target miR-455 and the protein of SOCS3 in the liver of db/db mice after intervention was significantly decreased. The dual luciferase reporter assay showed that SOCS3 was target gene of miR-455. In vitro, in Palmitate (PA)-stimulated human normal liver (LO2) cells, transfected miR-455 mimic could significantly inhibit the expression of SOCS3, while transfected miR-455 inhibitor could up-regulate the expression of SOCS3. Transfecting LO2 cells with siRNA of SOCS3 could significantly down-regulate the protein expression of SREBP-1c and ACCα. Our study showed that overexpression of miR-455 in the liver could improve lipid metabolism in diabetic mice by down-regulating its target gene SOCS3.

## Introduction

Non-alcoholic fatty liver disease (NAFLD) is closely related to diabetes. The estimated global prevalence of NAFLD is 25%, which is as high as 30% in some countries [1]. Recent clinical researches have revealed that diabetes plays an important role in the progression of NAFLD [2,3] and has become a strong predictor of non-alcoholic steatohepatitis (NASH) and liver fibrosis [4]. However, there is still a lack of effective treatment for NAFLD [5].

MicroRNA (miRNA) is a non-coding single-stranded RNA molecule of approximately 20–24 nucleotides in length, regulates gene expression at the post-transcriptional level by binding to the mRNA of the target gene [6]. MiRNA can regulate a variety of biological processes such as developmental processes, tumorigenesis and cell proliferation and apoptosis, not only participates in the process of multiple diseases, but also serves as a marker molecule for diagnosing diseases [7]. MiRNAs have been used as the clinical diagnostic markers for NAFLD/NASH, and even the potential predictors of primary liver cancer [8]. Our previous study shows that miR-455 is a key regulator of brown adipose tissue that promotes differentiation of brown adipocytes by targeting Necdin, Runx1t1 and HIF1an [9]. Recent studies indicate that miR-455 could improve the progression of different cancers [10,11], while there is still lack of the research focused on the regulation of miR-455 on liver metabolism.

In this study, we observed the changes in liver lipid metabolism in diabetic mice (db/db) after the overexpression of miR-455 in the liver. Furthermore, we found that miR-455 could improve the liver metabolism by inhibiting suppressor of cytokine signalling 3 (SOCS3), as a member of SOCS family, negatively regulates the transduction of cytokine signalling [12]. At the same time, SOCS3 also plays an important role in the regulation of metabolism. The overexpression of miR-19a [13] and miR-125a [14] could improve the metabolism of glucose and lipid through targeting SOCS3. We found that miR-455 ameliorated the lipid metabolic disorder of liver in db/db mice and this improvement may be caused by inhibition of SOCS3.

## Methods

### Animal experiments

All animal experiments were approved by the Animal Care and Use Committee of the Southern Medical University. 20 male C57BLKS/J diabetic mice (db/db; 6 weeks) and 20 male C57BLKS/J normal mice (db/m; 6 weeks) were purchased from the Nanjing Model Animal Centre. Mice were raised in the specific pathogen free barrier facility at the Southern Medical University under the controlled environment (temperature 22°C; 12 hours light/dark cycle). All mice were fed a standard chow diet (Guangzhou, China) and water.

After 1 week of adaptive culture, the mice were randomly divided into four groups: db/db-455 mice (*n* = 10), db/db-con mice (*n* = 10), db/m-455 mice (*n* = 10) and db/m-con mice (*n* = 10). The db/db-455 and db/m-455 mice were injected via tail vein with a plasmid construct expressing miR-455 (pCMV-miR-455)-miR-455 (Obio Technology, Shanghai) diluted with phosphate buffered saline and the db/db-con and db/m-con mice were given a pCMV (Obio Technology, Shanghai) injection. All mice received weekly injection for 4 weeks. When the experiment was over, the blood and liver tissue were collected after the mice were anesthetize by 1% pentobarbitol sodium.

### Metabolic measurement

The body weight (BW) and the random blood glucose (RBG) were recorded every 2 weeks. The serum and liver triglyceride (TG) content were detected by the Triglyceride detection kit (Jiancheng, Nanjing).

### Cell culture and transfection

Human normal liver cell line (LO2) and human embryonic kidney 293 cell line (HEK293) were obtained from the Cell Bank of the Chinese Academy of Sciences (Shanghai, China). All cells were cultured in Dulbecco’s Modified Eagle Medium (DMEM) medium (Gibco, USA) supplemented with 10% foetal bovine serum (FBS) (Gibco, USA) and 1% penicillin-streptomycin in a 5% CO2 atmosphere at 37°C. Palmitate (PA) (Sigma, USA) was used to mimic the hyperlipidaemia in cells. After stimulated with PA for 24 hours, the cells were transfected with miR-455 mimics, miR-455 inhibitors, small interfering RNAs (siRNAs) and negative control (RiboBio, Guangzhou) respectively for 48 hours, and the transfection was performed by Lipofectamine 3000 reagent (Invitrogen, MA).

### Bioinformatics prediction and dual luciferase reporter assay

TargetScan (http://www.targetscan.org/) was used to predict the potential targets of miR-455, the target site of miR-455-5p in SOCS3 3′-UTR was shown in Figure 4(g). Luciferase reporter plasmid wide-type (wt) or mutated-type (mut) were co-transfected into the 293 T cells with miR-455 mimics, respectively. The luciferase activity of the cells was measured by Luciferase Reporter Assay System (Promega Corporation) after 48 hours of transfection.

### Quantitative real-time PCR (qRT-PCR)

Total RNA was extracted from the cells and liver tissue by Trizol (Invitrogen, MA) and cDNA was synthesized by PrimeScript RT Master Mix (Takara, Japan). The cDNA of miR-455 and U6 was synthesized by MiRNA cDNA First Strand Synthesis Kit (TianGen, China). The qRT-PCR was performed with SYBR Green real-time Kit (Takara, Japan) and qRT-PCR of miR-455 and U6 was performed with SYBR Green real-time Kit (TianGen, China) on Roche LightCycler 480 Real-Time PCR System. β-actin was served as housekeeping gene in mouse liver and LO2 cells. U6 were used as housekeeping gene in compared with miR-455. Collect CT values of genes, and then use the 2‑∆∆Ct method to analyse the expression of all genes. All primers were listed in Tables 1 & 2.

**Table 1.** Primer sequences (mouse)

Gene	Forward prime (5ʹ-3ʹ)	Reverse prime (5ʹ-3ʹ)
β-actin	GTCCACCCCGGGGAAGGTGA	AGGCCTCAGACCTGGGCCATT
PPARγ	ATTCTGGCCCACCAACTTCGG	CTGCGGAAACTTCAGGAAATG
FAS	GGCCAGTGCTATGCTGAGAT	CACTTCTGGAGACATCGCAAAC
CD36	ATGGGCTGTGATCGGAACTG	TTTGCCACGTCATCTGGGTTT
SCD-1	CTGGCATTTGTTCCGGTTCT	CGGGATTGAATGTTCTTGTCGT
SREBP-1c	TGGAAGCCTGATGCTTTATCCCCA	GGTTCGGAATGCTATCCAGG
ACCα	TATTCGGCTGAAGCTGGTGTAC	TTCTTGCGATACACTCTGGTGC
TNF-α	CATCTTCTCAAAATTCGAGTGACAA	TGGGAGTAGACAAGGTACAACCC
MCP-1	GTTAACGCCCCACTCACCTG	GGGCCGGGGTATGTAACTCA
NOX4	TGCCTGCTCATTTGGCTGT	CCGGCACATAGGTAAAAGGATG
RUN6	TGGCCCCTGCGCAAGGATG	/
MiR-455-5p	TATGTGCCTTTGGACTACATCG	/

**Table 2.** Primer sequences (human)

Gene	Forward prime (5ʹ-3ʹ)	Reverse prime (5ʹ-3ʹ)
β-actin	CATGTACGTTGCTATCCAGGC	CTCCTTAATGTCACGCACGAT
PPARγ	GGGATCAGCTCCGTGGATCT	TGCACTTTGGTACTCTTGAAGTT
FAS	ATGTCAGTCACTTGGGCATTA	CATCTGGACCCTCCTACCTCT
ACCα	ATGTCTGGCTTGCACCTAGTA	CCCCAAAGCGAGTAACAAATTCT
SREBP-1c	CGGAACCATCTTGGCAACAGT	CGCTTCTCAATGGCGTTGT
PPARα	CTGGCATTTGTTTCTGTTCTTT	CTCCTCGGTGACTTATCCTGT
CPT-1	TCCAGTTGGCTTATCGTGGTG	TCCAGAGTCCGATTGATTTTTGC
IL-6	ACTCACCTCTTCAGAACGAATTG	CCATCTTTGGAAGGTTCAGGTTG
TNF-α	CCTCTCTCTAATCAGCCCTCTG	GAGGACCTGGGAGTAGATGAG
NOX4	CAGATGTTGGGGCTAGGATTG	GAGTGTTCGGCACATGGGTA

### Western blot analysis

The protein of cells and liver tissue was extracted by RIPA lysis buffer (KeyGen, China) and the concentration of protein was quantified by BCA Protein Assay Kit (Takara, Japan). The denatured protein (20 ug) was separated by 10% sodium dodecyl sulphate-polyacrylamide gel electrophoresis and transferred onto the polyvinylidene difluoride membrane. After being blocked with 5% skim milk for 1 hour at ambient temperature, the membrane was incubated with primary antibodies of sterol regulatory element binding protein 1c (SREBP-1c) (1:1000, Abclone, China), acetyl-CoA carboxylase (ACCα) (1:1000, Abclone, China), fatty acid synthase (FAS) (1:1000, Abclone, China), NADPH (nicotinamide adenine dinucleotide phosphateoxidase) oxidase 4 (NOX4) (1:1000, Abclone, China), SOCS3 (1:1000, Abclone, China), IL-6 (1:1000, CST, USA) and β-actin (1:1000, Zhongshan, China) at 4°C overnight. Then the membrane was incubated with horseradish peroxidase labelled secondary antibody (1:5000) for 1 hour after being washed three times with TBST (tris-buffered saline Tween-20). Enhanced chemiluminescence reagent (Millipore, USA) was used to detect immunoreactive bands and the Image J software was used to analyse the bands.

### Histological staining

The liver tissue was first fixed with 4% paraformaldehyde, then dehydrated and embedded in paraffin. The tissue embedded in the paraffin was subjected to H&E staining, and the freshly fixed tissue was stained with oil red O. Olympus B × 20 upright light microscope (Olympus, Tokyo, Japan) was used to detect all sections.

### Statistical Analysis

Data were expressed as the mean ± SD. Two-tailed Student’s *t* test was used to compare two independent groups, and one-way ANOVA was used to examine the differences among four independent groups. Statistical analysis was performed by using SPSS 20.0. *P* < 0.05 was considered statistically significant.

## Results

### The changes of basal metabolic indicator in mice after the intervention of miR-455

**Figure 1.**
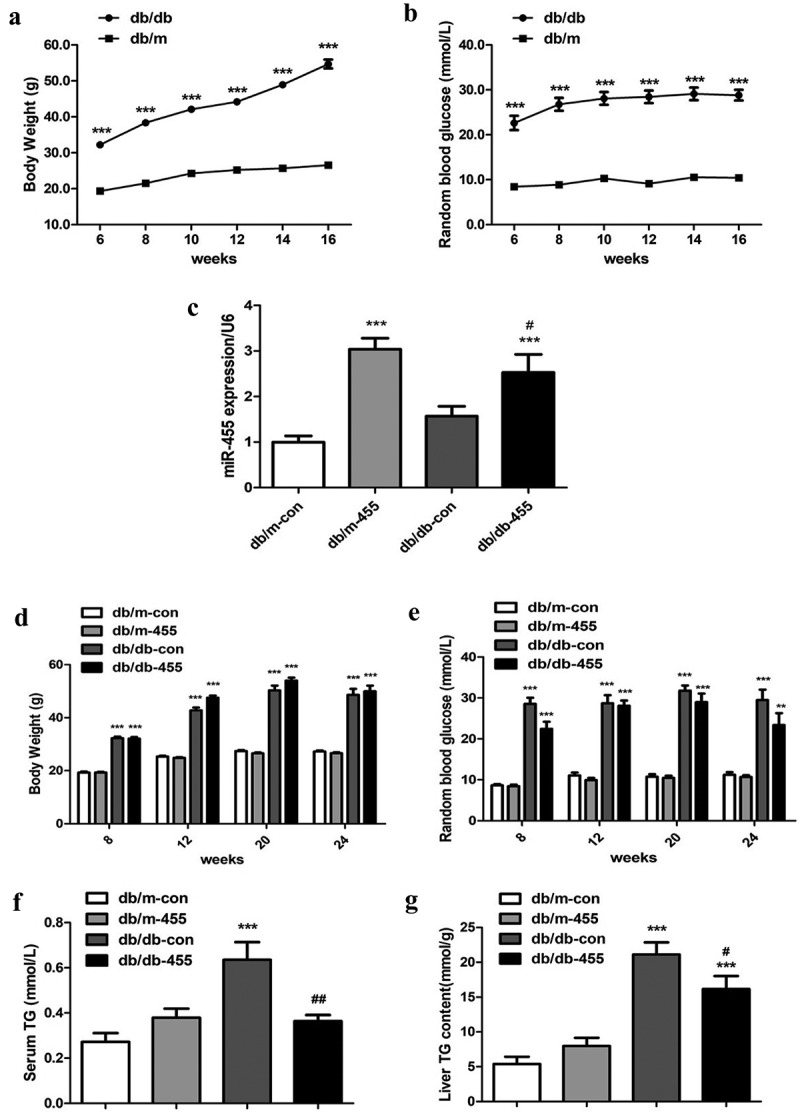
The changes of basic metabolism indicator of mice after intervention by miR-455. The BW (g) (a) and RBG (mmol/L) (b) of mice before intervention. (C) (c) The expression of miR-455 in liver of mice. The BW (d) and the RBG (e) of mice after intervention. The serum (f) and liver TG (g) content of mice. *n* = 6–8 per group

Db/db mice showed obvious obesity after 6 weeks and polydipsia, polyphagia and polyuria. The weight (Figure 1(a)) and RBG (Figure 1(b)) of db/db mice were also significantly higher than those of db/m mice, indicating the establishment of diabetes model. After intervention, the expression of miR-455 was significantly increased in the liver of db/db-455 mice and db/m-455 mice compared to db/db-con mice and db/m-con mice respectively (Figure 1(c)). During the whole experiment, the BW (Figure 1(d)) and RBG (Figure 1(e)) of db/db-con and db/db-455 mice were higher than those of db/m mice. Although there was no obvious improvement in the levels of BW and blood glucose in db/db-455 mice, the overexpression of miR-455 in liver significantly reduced serum TG levels (Figure 1(f)) and liver TG content (Figure 1(g)) compared with db/db-con mice.

### Overexpression of miR-455 improved the liver lipid metabolism

**Figure 2.**
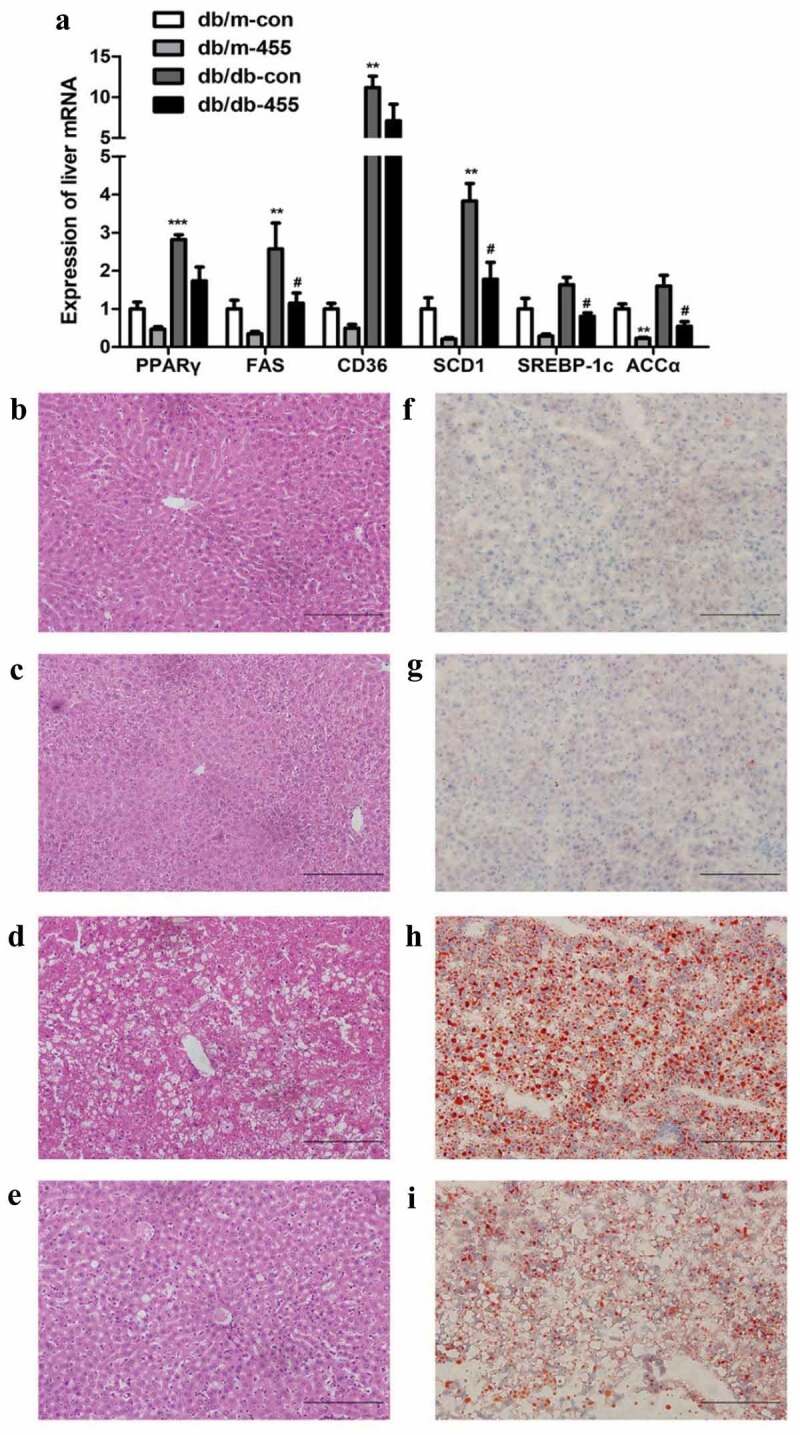
Overexpression of miR-455 improved the liver lipid metabolism. (a) The mRNA expression of PPARγ, FAS, CD36, SCD-1,SREBP-1c and ACCα in the liver of mice in four groups. H&E (b–e) and oil red O staining (f–i) of liver in four groups. Db/m-con group (b + f), db/m-455 group (c + g), db/db-con group (d + h), db/db-455 group (e + i). Magnification ×400. *n* = 6–8 per group. **P < 0.01 and ***P < 0.001 vs db/m-con group; #*P* < 0.05 vs db/db-con group

**Figure 3.**
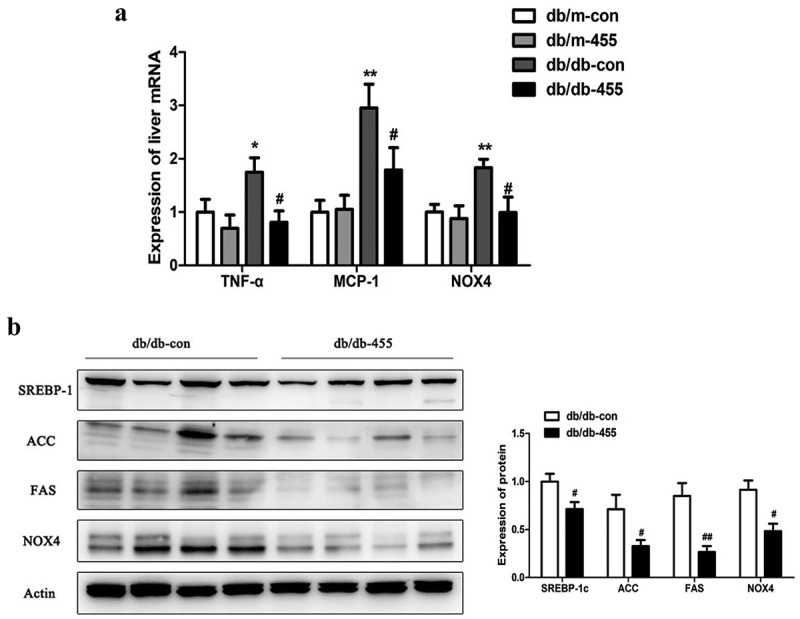
Overexpression of miR-455 ameliorated the liver inflammation and oxidative stress. (a): The mRNA expression of TNF-α, MCP-1 and NOX4 of liver in four groups. (b) The protein levels of SREBP-1c, ACC, FAS and NOX4 in liver of db/db-con group and db/db-455 group, the quantitative analysis results of protein levels. *n* = 6–8 per group. *P < 0.05 and **P < 0.01 vs db/m-con group; #*P* < 0.05 and ##*P* < 0.01 vs db/db-con group

Compared with db/m-con mice, the mRNA expression of peroxisome proliferator-activated receptor γ (PPARγ), FAS, fatty acid transporter (CD36) and stearoyl-CoA desaturase 1 (SCD-1) was notable up-regulated in db/db-con mice, the mRNA expression of ACCα and SREBP-1c also increased although without statistical significant (Figure 2(a)). However, the mRNA expression levels of FAS, SCD-1, SREBP-1c and ACCα in db/db-455 mice were significantly lower than those in db/db-con group (Figure 2(a)). In addition, we detected the protein expression of SREBP-1c, ACCα and FAS in the liver of db/db-455 and db/db-con mice, the results were consistent with the expression trend of mRNA (Figure 3(b)). H&E staining showed liver structural disorder, vacuolar degeneration of hepatocytes and inflammatory infiltration in db/db-con mice (Figure 2(d)) compared with db/m-con mice (Figure 2(b)), but the overexpression of miR-455 in liver ameliorated these changes in db/db-455 mice (Figure 2(e)). Simultaneously, the oil red O staining revealed that lipid deposition in liver of db/db-con mice (Figure 2(h)) was severe than db/m-con mice (Figure 2(f)), while the overexpression of miR-455 significantly reduced the number and size of liver lipid droplets in db/db-455 mice (Figure 2(i)).

### Overexpression of miR-455 ameliorated the liver inflammation and oxidative stress

We then detected the liver mRNA expression of tumour necrosis factor-α (TNF-α), monocyte chemotactic protein-1 (MCP-1) and NADPH oxidase 4 (NOX4), as the indicators of inflammation, the high expression of TNF-α and MCP-1 in db/db-con mice was significantly reduced after miR-455 overexpression in the liver of db/db-455 mice (Figure 3(a)). Overexpression of miR-455 in liver also tremendously inhibited the mRNA expression of NOX4 in db/db-455 mice, an indicator of oxidative stress (Figure 3(a)). Also, the protein expression level of SREBP-1c, ACCα, FAS and NOX4 was significantly decreased in the liver of db/db-455 mice compared with db/db-con mice (Figure 3(b)).

### Cytokine signal transduction inhibitor 3 (SOCS3) was the target gene of miR-455 in liver

**Figure 4.**
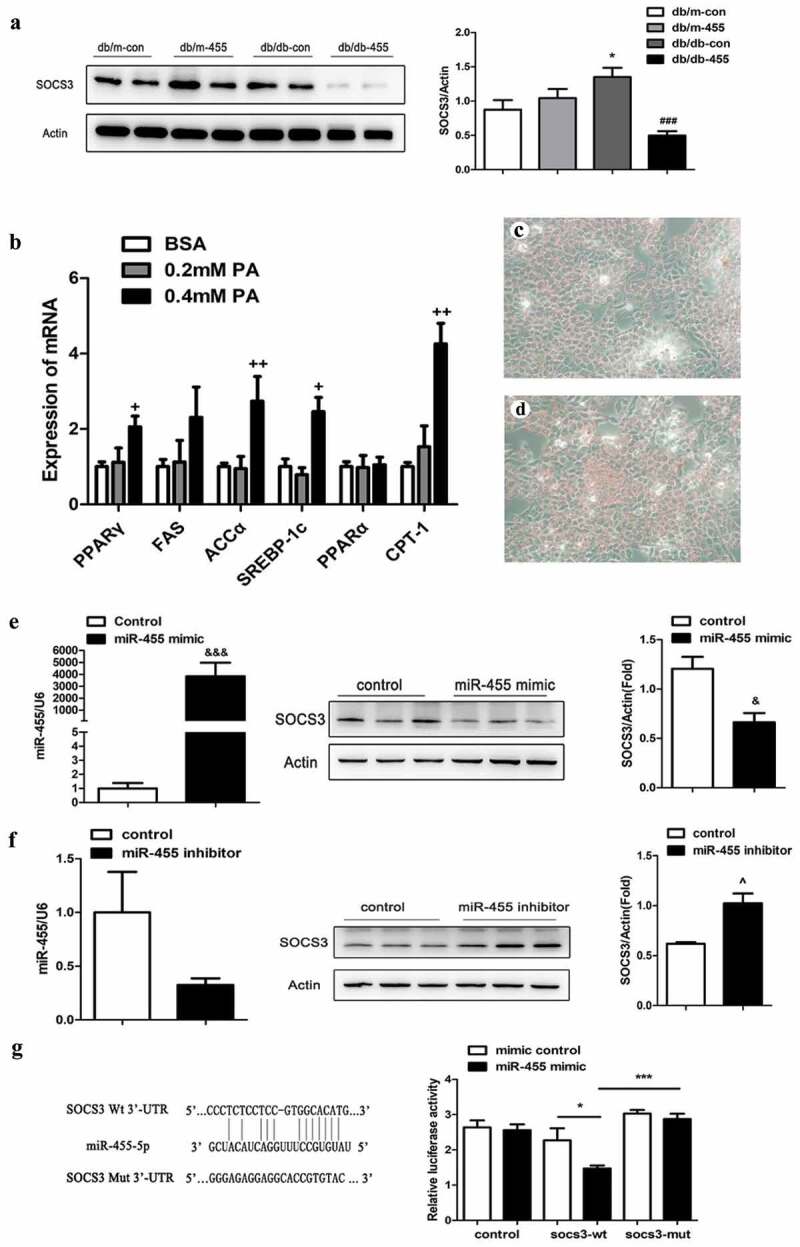
SOCS3 was the target gene of miR-455 in liver. (a) The protein level of SOCS3 in liver in four groups and the quantitative analysis result (*n* = 6–8 per group). (b) The mRNA expression of PPARγ, FAS, SREBP-1c, ACCα, PPARα and CPT-1 in LO2 cells. The oil red O staining of cells stimulated with BSA (bovine serum albumin) (c) and 0.4 mM PA (d). (e) The expression of miR-455 and SOCS3 in LO2 cells after treatment of miR-455 mimic. (f) The expression of miR-455 and SOCS3 in LO2 cells after treatment of miR-455 inhibitor. (g) The binding sites of miR-455 and SOCS3. Luciferase reporter assay of the interaction between miR-455 and SOCS3 (*n* = 3 per group). *P < 0.05 vs db/m-con group; ###*P* < 0.001 vs db/db-con group. +*P* < 0.05 and ++ *P* < 0.01 vs BSA group. &*P* < 0.05 and &&&*P* < 0.001 vs mimic control group. ^*P* < 0.05 vs inhibitor control group. *P < 0.05 and ***P < 0.001

Since the overexpression of miR-455 could improve the lipid metabolism, inflammation and oxidative stress of db/db mice, we predicted its possible target genes using TargetScan software in order to investigate its underlying mechanism. We found that SOCS3 may be one of the target genes of miR-455, and the detail bioinformatic analysis for the target site of miR-455-5p in SOCS3 3′-UTR was shown in Figure 4(g). Next, we detected the protein levels of SOCS3 in mice. The result indicated that the overexpression of miR-455 in liver obviously inhibited the expression of SOCS3 in db/db-455 mice (Figure 4(a)).

To mimic the hyperlipidaemia in vitro, we incubated the LO2 cells with PA. Lipid metabolism-related gene expression levels were significantly higher at 0.4 mM PA stimulation than 0.2 mM PA (Figure 4(b)), so we choose a stimulus concentration of 0.4 mM and the oil red O staining also indicated the 0.4 mM was a suitable concentration (Figure 4(c,d)). On the basis of PA stimulation, cells were transfected with mimic and inhibitor of miR-455 respectively. The expression of miR-455 in LO2 cells increased significantly after treatment with miR-455 mimic (Figure 4(e)), also the expression of miR-455 decreased after intervention with miR-455 inhibitor (Figure 4(f)). The protein level of SOCS3 was remarkably down-regulation after intervention by miR-455 mimic in LO2 cells (Figure 4(e)). On the contrary, the protein level of SOCS3 was significantly up-regulated after intervention by miR-455 inhibitor (Figure 4(f)). Then we constructed the SOCS3 wt plasmids and mut plasmids, which were co-transfected into 293 T cells with miR-455 mimic. The luciferase assay showed that the luciferase activity was significantly decreased after co-transfection of miR-455 mimic and SOCS3 wt plasmids compared to co-transfection with miR-455 mimic and SOCS3 mut plasmids (Figure 4(g)). These results demonstrated that SOCS3 was a target gene of miR-455 in liver.

### The effect of knocking down the expression of SOCS3 in LO2 cells

**Figure 5.**
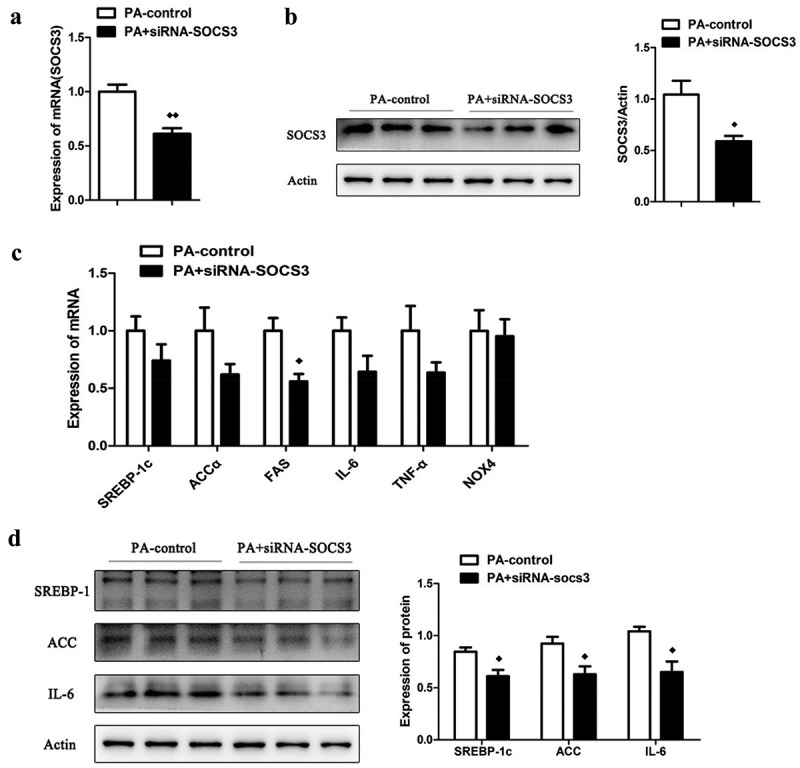
The effect of knocking down the expression of SOCS3 in LO2 cells. The mRNA (a) and protein (b) expression of SOCS3 in LO2 cells after incubated with PA and siRNA-SOCS3. (c) The mRNA expression of SREBP-1c, ACCα, FAS, IL-6, TNF-α and NOX4 in LO2 cells after incubated with PA and siRNA-SOCS3. (d) The protein expression of SREBP-1c, ACCα and IL-6 in cells after c results. *n* = 3 per group. ◆◆*P* < 0.05 and ◆◆◆◆*P* < 0.01 vs PA-control group

In order to investigate whether knocking down SOCS3 could affect the metabolism of LO2 cells, we treated the LO2 cells with siRNA-SOCS3 on the basis of PA. The mRNA (Figure 5(a)) and protein (Figure 5(b)) expression of SOCS3 was tremendously down-regulation after siRNA-SOCS3 intervention. SiRNA intervention significantly reduced mRNA expression of FAS in cells (Figure 5(c)). Despite the mRNA expression of SREBP-1c, ACCα, TNF-α and interleukin-6 (IL-6) had a decreasing trend, it had not reached a significant difference. Nevertheless, siRNA intervention of SOCS3 significantly decreased the protein expression of SREBP-1c, ACCα and IL-6 (Figure 5(d)).

## Discussion

The synthesis of hepatic TGs exceeds the rate of catabolism could lead to the accumulation of TGs. And excessive accumulation of TGs in hepatocytes contribute to the occurrence of NAFLD [15]. In our study, we observed obvious obesity and lipid deposition in liver of db/db mice consistent with the result of published studies [16,17]. MicroRNAs play an important role in a variety of diseases [18–20] including the lipid metabolic disorder [21,22], because they can regulate a wide range of growth and development processes [7]. Recent studies indicate that miR-27a [23], miR-194 [24] and miR-146b [25] can target different genes to regulate the development of NAFLD. According to our previous research, miR-455 is the key regulator of BAT in the process of differentiation [9] and may have an effect on the regulation of lipid synthesis. Then, we achieved the overexpression of miR-455 to figure out whether it could improve the lipid metabolism of liver in db/db mice. The results showed that the serum and liver TG content of db/db-455 mice was significantly decreased. Histological staining results also suggested that there was improvement in the structural disorder and lipid deposition in the liver of db/db-455 mice.

Liver is a central regulator of lipid metabolism in human [26]. Nowadays, a lot of researches have already confirmed that plenty of molecules are involved in the lipid metabolism, including PPARγ, SREBP-1c, FAS and ACCα [26–28]. As the crucial genes for the development of NAFLD, these genes were significantly up-regulated in the liver of db/db mice. There was also a significant increase in CD36 expression, which involved in fatty acid transport [29]. However, increased expression of these genes was conspicuously inhibited after the overexpression of miR-455 in liver. SREBP-1c has been reported as a upstream signal to directly regulate ACCα and FAS [30]. In our study, overexpression of miR-455 suppressed the protein levels of SREBP-1c, ACCα and FAS. Thus, miR-455 could ameliorate the lipid deposition in db/db mice by downregulating SREBP-1c and its downstream signal pathway.

SOCS3 has been reported involving in the progress of various diseases such as autoimmune diseases [31], cardiovascular diseases [32], metabolic diseases [33] and tumours [34], which is responsive to an increase in IL-6 and plays a negative regulatory role in cytokine signalling [12]. It has been shown that miR-19a-3p can promote cell proliferation and insulin secretion by targeting SOCS3 [13], overexpression of miR-125a-5p also inhibit the expression of SOCS3 to improve glycolipid metabolism in the liver [14]. In our study, miR-455 can significantly reduce serum and liver TG level without affecting BW and blood glucose, the probable reason is that the cycle of the experiment is not long enough to significantly improve blood glucose. In the end of the animal experiment, we found that the RBG level of db/db-455 group has been decreasing, but there was no significant difference compared with db/db-con group. We confirmed that SOCS3 was a target gene of miR-455. And Inhibition of SOCS3 could down-regulate SREBP-1c, ACCα and FAS in LO2 cells incubated with PA. Studies have shown that overexpression of SOCS3 enhances the promoter activity of SREBP-1c and then promotes the transcription of SREBP-1c [35]. Therefore, combined with our in vitro and in vivo experiments, we considered that overexpression of miR-455 can regulate lipid synthesis genes such as SREBP-1c by inhibiting its target gene SOCS3, thereby improve liver lipid metabolism.

Liver inflammation and oxidative stress are very important mechanisms of NAFLD [36]. In our study, we observed a significant increase of TNF-α, MCP-1 and NOX4 in db/db mice, which were significantly decreased by overexpression of miR-455 in db/db mice. In vitro, IL-6 was further decreased by transfecting siRNA of SOCS3 in LO2 cells. It has been reported that SOCS3 could promote the expression of inflammatory factors by inhibiting the phosphorylation of JAK2/STAT3 signalling pathway in adipocytes [37].

In conclude, we found that overexpression of miR-455 in db/db mice could improve the lipid metabolism, inflammation and oxidative stress in liver by targeting SOCS3. MiR-455/SOCS3 pathway may be a potential target for the treatment of abnormal liver lipid metabolism in type 2 diabetes.

## Data Availability

The analysed data sets generated during this study are available from the corresponding author on reasonable request.
